# Supplementary material for: Antifungal Hybrid Graphene–Transition-Metal Dichalcogenides Aerogels with an Ionic Liquid Additive as Innovative Absorbers for Preventive Conservation of Cultural Heritage
Source: Materials (Basel). 2024 Jun 28;17(13):3174. doi: 10.3390/ma17133174 (PMC11242601; doi:10.3390/ma17133174)
Supplement: Supplementary file 1 [file materials-17-03174-s001.zip › materials-3066176-supplementary.pdf]

Antifungal Hybrid Graphene–Transition-Metal Dichalcogenides Aerogels with an Ionic Liquid Additive as Innovative Absorbers for Preventive Conservation of Cultural Heritage

George Gorgolis [1,2,*], Maria Kotsidi [2], Elena Messina [3], Valentina Mazzurco Miritana [4], Gabriella Di Carlo [3,*], Elsa Lesaria Nhuch [5], Clarissa Martins Leal Schrekker [5], Jeniffer Alves Cuty [5], Henri Stephan Schrekker [5,*], George Paterakis [1], Charalampos Androulidakis [6], Nikos Koutroumanis [2] and Costas Galiotis [1,2,*]

1 Institute of Chemical Engineering Sciences, Foundation of Research and Technology-Hellas (FORTH/ICE-HT), Stadiou Street, Platani, 26504 Patras, Greece

2 Department of Chemical Engineering, University of Patras, 26504 Patras, Greece

3 Institute for the Study of Nanostructured Materials (ISMN), National Research Council (CNR), SP35d, 9, 00010 Montelibretti, Italy; elena.messina@cnr.it

4 Department of Energy Technologies and Renewable Sources, Italian National Agency for New Technologies, Energy and Sustainable Economic Development (ENEA), Via Anguillarese 301, 00123 Rome, Italy

5 Laboratory of Technological Processes and Catalysis, Institute of Chemistry, Federal University of Rio Grande do Sul, Av. Bento Gonçalves 9500, Porto Alegre 91.501-970, RS, Brazil

6 Skeletal Biology and Engineering Research Center, Department of Development and Regeneration, KU Leuven, O&N1, Herestraat 49, PB 813, 3000 Leuven, Belgium

* Correspondence: ggorgolis@iceht.forth.gr (G.G.); gabriella.dicarlo@cnr.it (G.D.C.); henri.schrekker@ufrgs.br (H.S.S.); c.galiotis@iceht.forth.gr (C.G.)

Synthesis of neat rGO aerogels

An aqueous solution of GO was prepared by modified Hummer's method [1], [2] and was subsequently diluted in water to obtain a concentration of 1 mg/ml. At first, the starting GO sheets are randomly dispersed in water due to their strong hydrophilicity and electrostatic repulsion. Hypophosphorous acid (H_3PO_2) and iodine (I_2) of weight ratio GO: H_3PO_2:I_2 equal to 1:100:10 were added as the reducing agents [3] (step i). Following that, the prepared solutions were placed in a furnace at 80°C for 8 hours (step ii), resulting in the gelation of GO (step iii). The gelation mechanism involves the simultaneous reduction (H_3PO_2 reacts with I_2 to form hydriodic acid, which works as a reducing agent and is responsible for the pH reduction of the medium to lower than 1) and self-assembly of the rGO sheets [3]. During the reduction of graphene oxide solution, the GO sheets become regionally hydrophobic due to the removal of oxygen functional groups. The hydrogen bonding with water becomes weakened, and the van der Waals attraction between the planes increases, creating some sticky graphene sheets. The combination of hydrophobic and π-π interactions leads to a 3D random stacking between flexible graphene sheets. With the reaction proceeding, the hydrophobicity and the π-conjugated structures of reduced GO sheets are increased, which enhances the π-π stacking of GO sheets to a compact and rigid structure [4], [5]. The formed hydrogels take the shape of the containment vessel. Following the gelation process, the formed hydrogels were rinsed thoroughly with distilled water until a pH of 6-7 was reached

and pre-freezed at -27°C for up to a week. Finally, the frozen samples were freeze-dried (Telstar Cryodos freeze-dryer) for 48 hours (step iv) to form the rGO aerogels (step v).

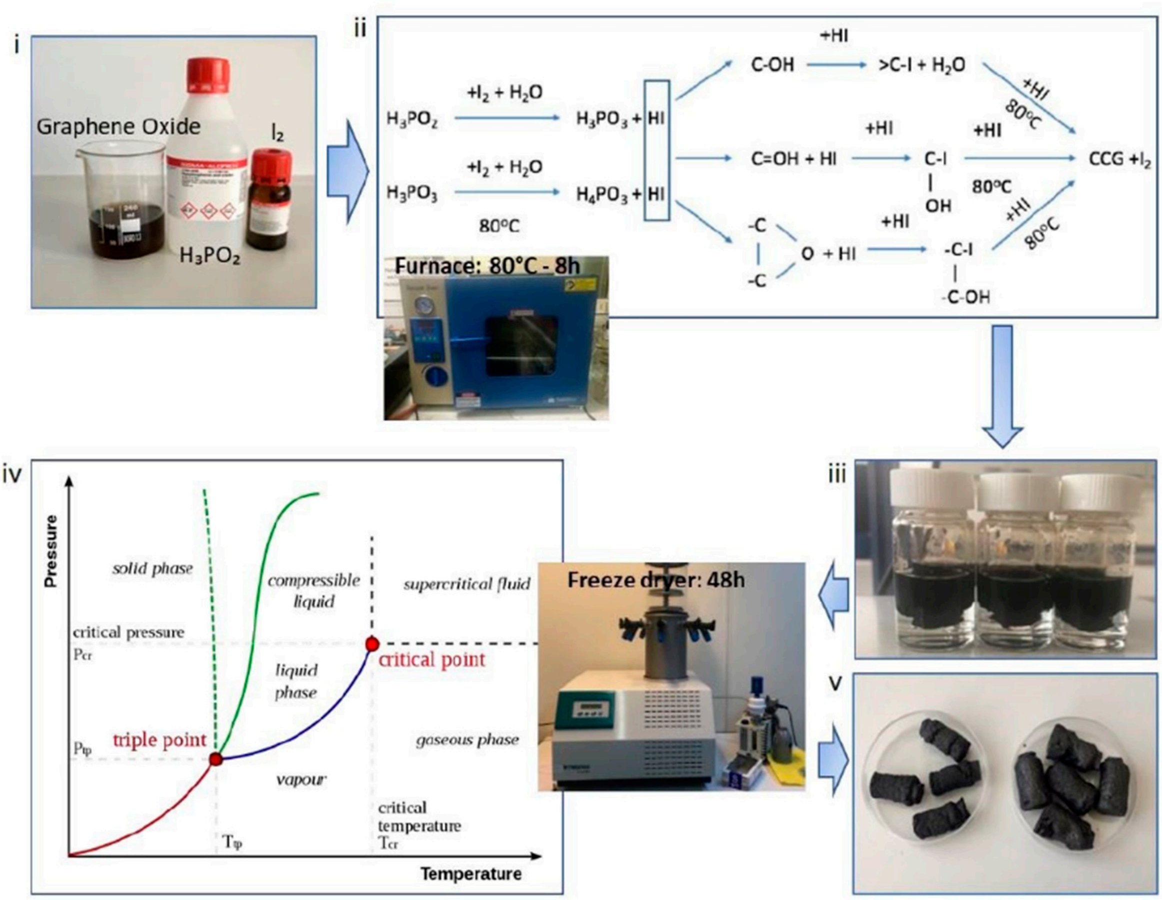

Figure S1: Schematic representation of the experimental process of rGO aerogels production. In step (II), the chemical reduction of GO using H_3PO_2 and I_2 as reducing agents is presented [6], and in step (iv), the water phase diagram is shown [7].

<u>Volatile organic compounds (VOCs) absorption tests</u>

100 ml of each VOC: formaldehyde (37 wt.% in H_2O), acetic acid (99 wt.% in H_2O), formic acid (85 wt.% in H_2O) or ammonia (30 wt.% in H_2O) was used as the pollutant source each time. It was found that each pollutant created different relative humidity (RH) conditions inside the desiccator; in particular, CH_2O resulted in 75% relative humidity, CH_2O_2 in 99%, CH_3COOH and NH_3 in 55%. All samples were initially dried at 200°C for two hours to remove the absorbed humidity and

weighed into a high accuracy weight meter to measure their dried mass. Afterwards, the aerogels were loaded in a glass Petri dish which was mounted inside the desiccator. The desiccator with the aerogels and the fuming gas was constantly inside a fume hood. Periodically, the mass of each sample was recorded by the weight meter, exactly next to the fume hood minimizing the exposure of the aerogels to the environment.

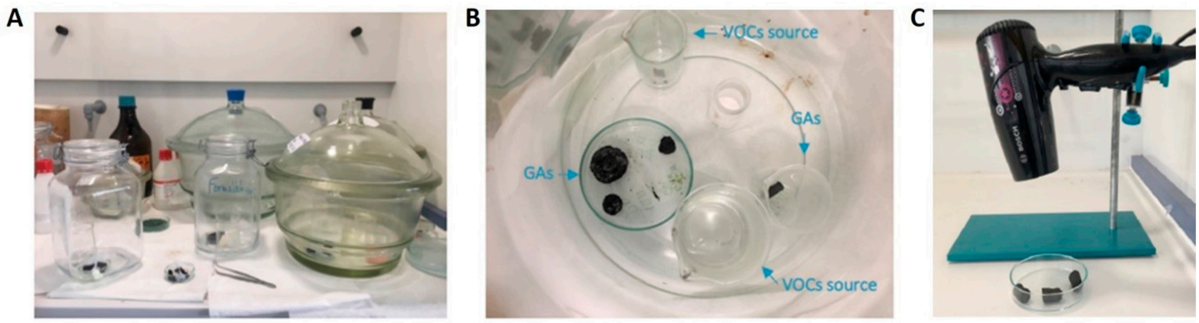

Figure S2: (A, B) Experimental set-up for the measurement of VOCs absorption from the prepared graphene aerogels. (C) Regeneration of aerogels using an electric hairdryer (an aluminum foil with tiny holes was also used to cover the petri dish in order for the aerogels not to escape).

XPS-rGO/ionic liquid

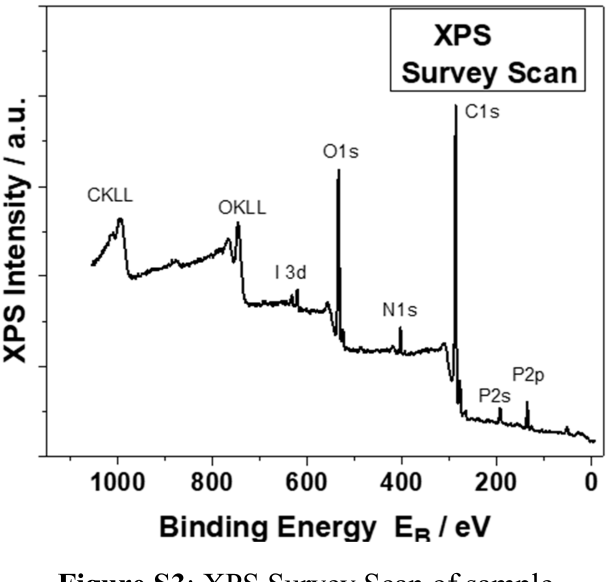

Figure S3: XPS Survey Scan of sample

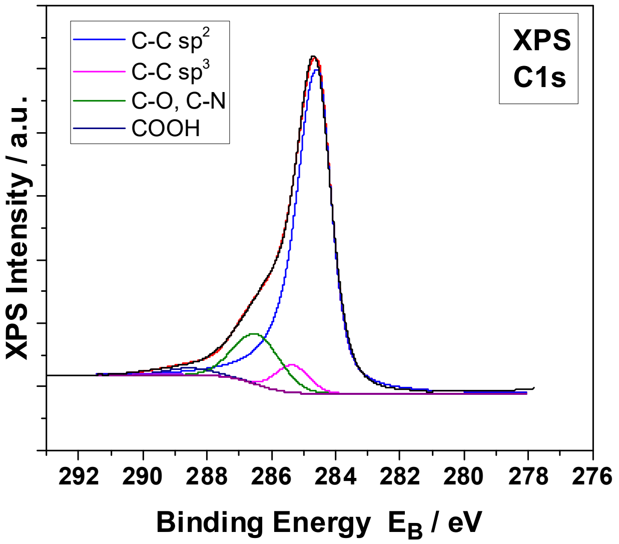

Figure S4: XPS Deconvoluted C1s of sample

Table S1: Quantification

Peak	Eb [eV]	assignement	%at. concentration
**O1s**	532.37	C=O, O-P-O [8]	17.93 ± 0.03
**C1s**	284.49	C-C sp^2	73.46 ± 0.04
**P2p**	133.46	P^{5+} (P_2O_5) [8]	5.63 ± 0.03
**I3d**	618.93	I^- [9]	0.35 ± 0.00
**N1s**	401.57	Graphitic Nitrogen [10] or C-NH_2 [11]	2.62 ± 0.02

Taking into account the presence of P_2O_5 compound, we estimate that ~14% oxygen atoms are bonded with phosphorous (5.6*(5/2) =14). Thus, ~4% of oxygen atoms are bonded with carbon and the O/C ratio is 0.05.

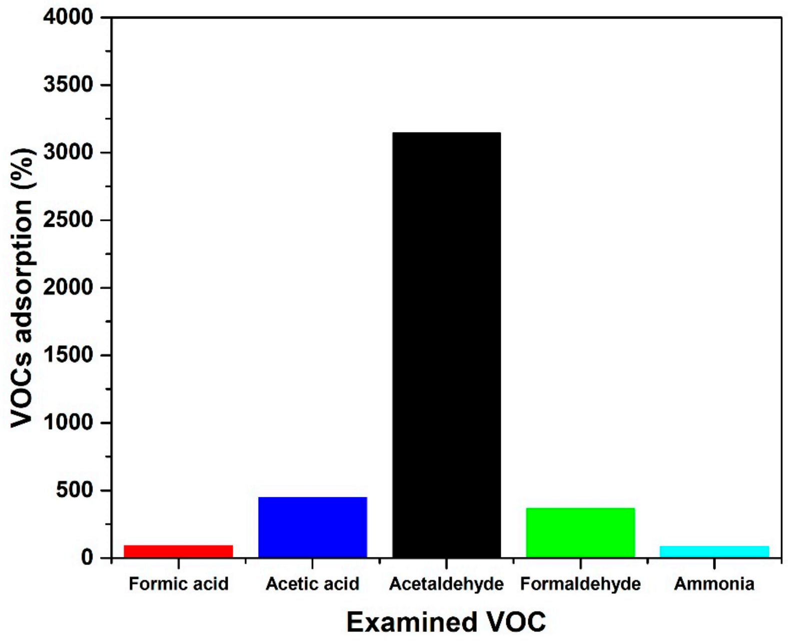

Figure S5: VOCs absorption results for neat rGO aerogel sample.

Table S2: Maximum absorption capacity of commercial absorption materials (activated carbon, silica gel and polyurethane) for the examined VOCs

	Maximum absorption capacity (%)		
VOC	Activated carbon	Silica gel	polyurethane
Formaldehyde	29	19	56
Ammonia	16	13	44
Acetic acid	9	20	9
Formic acid	15	14	7

Table S3: Maximum absorption capacity of commercial absorption materials (activated carbon, silica gel and polyurethane) for the examined RH conditions

	Maximum absorption capacity (%)		
RH	Activated carbon	Silica gel	polyurethane
55% (ammonia and acetic acid)	6.5	10	1
75% (formaldehyde)	10	10.5	2
99% (formic acid)	17	28	6.5

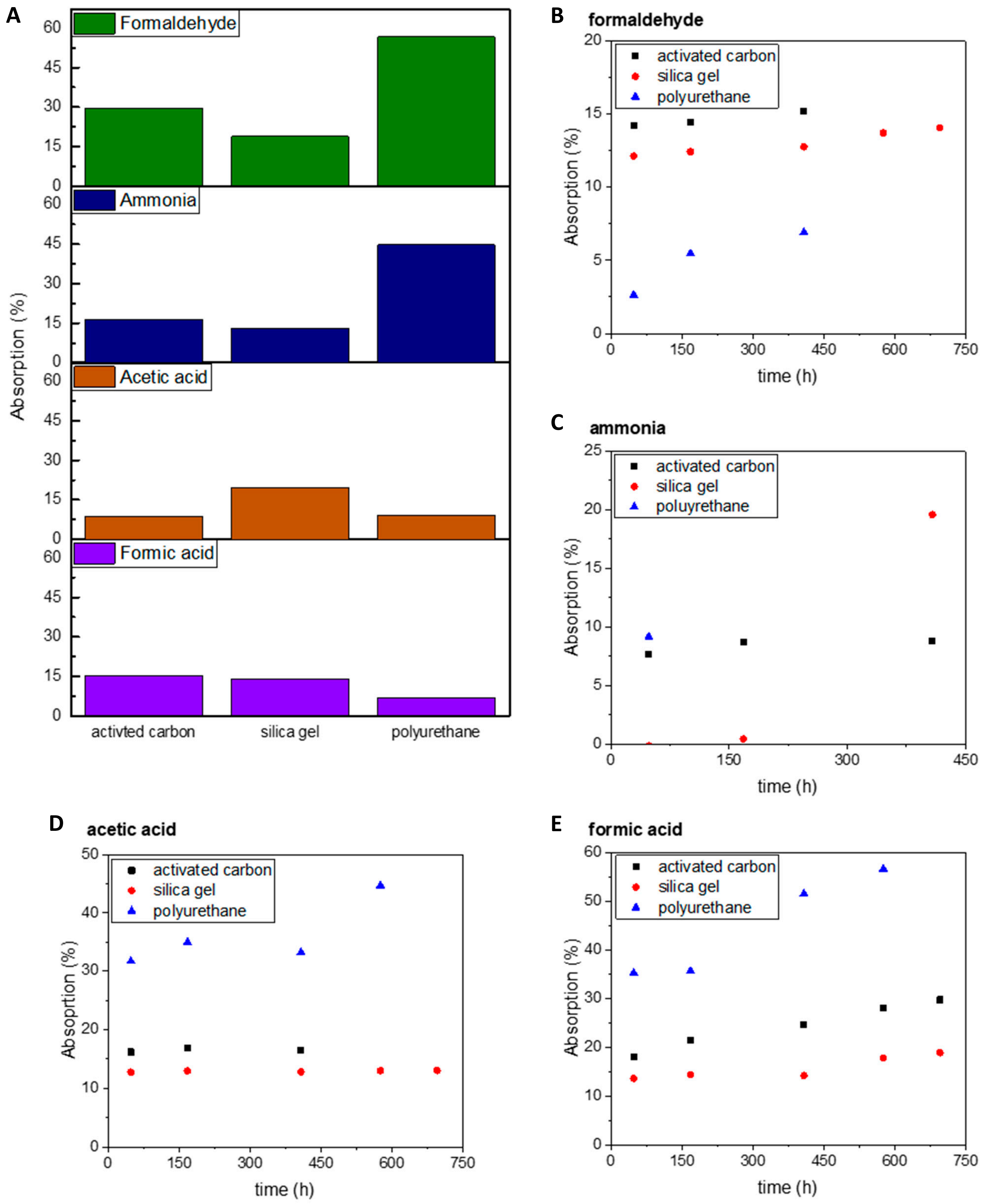

Figure S6: (A) Maximum absorption capacity of commercial absorption materials (activated carbon, silica gel and polyurethane) for the examined VOCs. (B-E) Kinetics of absorption of commercial absorption materials for the examined VOCs.

REFERENCES

[1] W. S. Hummers and R. E. Offeman, “Preparation of Graphitic Oxide,” *J. Am. Chem. Soc.*, vol. 80, no. 6, p. 1339, 1958, doi: 10.1021/ja01539a017.

[2] N. I. Kovtyukhova, P. J. Ollivier, B. R. Martin, T. E. Mallouk, E. V. Buzaneva, and A. D. Gorchinskiy, “Layer-by-layer assembly of ultrathin composite films from micron-sized graphite oxide sheets and polycations,” *Chem. Mater.*, vol. 11, no. 3, pp. 771–778, 1999, doi: 10.1021/cm981085u.

[3] H. D. Pham *et al.*, “Synthesis of the chemically converted graphene xerogel with superior electrical conductivity,” *Chem. Commun.*, vol. 47, no. 34, pp. 9672–9674, 2011, doi: 10.1039/c1cc13329b.

[4] W. Tang *et al.*, “Facile synthesis of 3D reduced graphene oxide and its polyaniline composite for super capacitor application,” *Synth. Met.*, vol. 202, pp. 140–146, 2015, doi: 10.1016/j.synthmet.2015.01.031.

[5] V. Chabot, D. Higgins, A. Yu, X. Xiao, Z. Chen, and J. Zhang, “A review of graphene and graphene oxide sponge: Material synthesis and applications to energy and the environment,” *Energy Environ. Sci.*, vol. 7, no. 5, pp. 1564–1596, 2014, doi: 10.1039/c3ee43385d.

[6] T. Pham *et al.*, “Nanoscale structure and superhydrophobicity of sp2-bonded boron nitride aerogels,” *Nanoscale*, vol. 7, no. 23, pp. 10449–10458, 2015, doi: 10.1039/c5nr01672j.

[7] Pipolo, S.; Salanne, M.; Ferlat, G.; Klotz, S.; Saitta, M.; Pietrucci, F. Navigating at Will on the Water Phase Diagram. *Phys. Rev. Lett.* **2017**, *119*, 245701.

[8] A. Thøgersen, M. Syre, B. Retterstol Olaisen, and S. Diplas, “Studies of the oxidation states of phosphorus gettered silicon substrates using X-ray photoelectron spectroscopy and transmission electron microscopy,” *J. Appl. Phys.*, vol. 113, no. 4, 2013, doi: 10.1063/1.4775818.

[9] K. Siuzdak, M. Szkoda, M. Sawczak, A. Lisowska-Oleksiak, J. Karczewski, and J. Ryl, “Enhanced photoelectrochemical and photocatalytic performance of iodine-doped titania nanotube arrays,” *RSC Adv.*, vol. 5, no. 62, pp. 50379–50391, 2015, doi: 10.1039/c5ra08407e.

[10] V. Deerattrakul, N. Yigit, G. Rupprechter, and P. Kongkachuichay, “The roles of nitrogen species on graphene aerogel supported Cu-Zn as efficient catalysts for CO2 hydrogenation to methanol,” *Appl. Catal. A Gen.*, vol. 580, no. January, pp. 46–52, 2019, doi: 10.1016/j.apcata.2019.04.030.

[11] J. Wu, W. Wang, and Z. Wang, “Porphin-based carbon dots for ‘Turn off–on’ phosphate sensing and cell imaging,” *Nanomaterials*, vol. 10, no. 2, 2020, doi: 10.3390/nano10020326.
